# A framework for integrating GWAS and single-cell RNA-seq with healthy reference controls to identify disease-associated cells

**DOI:** 10.1093/bib/bbaf631.071

**Published:** 2025-12-12

**Authors:** Caicai Zhang, Frank Qingyun Wang, Xiao Dang, Huidong Su, Xingtian Yang, Yao Lei, Youming Guo, Xinxin Chen, Wanling Yang

**Affiliations:** Department of Paediatrics and Adolescent Medicine, The University of Hong Kong, Hong Kong, China; Department of Paediatrics and Adolescent Medicine, The University of Hong Kong, Hong Kong, China; Department of Paediatrics and Adolescent Medicine, The University of Hong Kong, Hong Kong, China; Department of Paediatrics and Adolescent Medicine, The University of Hong Kong, Hong Kong, China; Department of Paediatrics and Adolescent Medicine, The University of Hong Kong, Hong Kong, China; Department of Paediatrics and Adolescent Medicine, The University of Hong Kong, Hong Kong, China; Department of Paediatrics and Adolescent Medicine, The University of Hong Kong, Hong Kong, China; Department of Paediatrics and Adolescent Medicine, The University of Hong Kong, Hong Kong, China; Department of Paediatrics and Adolescent Medicine, The University of Hong Kong, Hong Kong, China

## Abstract

**Background:**

Genome-wide association studies (GWAS) have revealed numerous loci for complex diseases, yet the cellular contexts through which these variants act remain poorly understood. Single-cell RNA sequencing (scRNA-seq) offers high-resolution profiling of cellular heterogeneity, but linking GWAS signals to disease-relevant cells remains a challenge.

**Methods:**

We developed a statistical framework that integrates GWAS-prioritized genes with single-cell transcriptomes to detect disease-relevant cells. Each target cell is contrasted with pools of healthy reference cells, ensuring that differences reflect disease rather than technical variability. Two controls are implemented: reference cells matched by sequencing depth and expression-matched control genes without known disease associations. Iterative Monte Carlo sampling generates comparisons across random draws of reference cells and control genes, reducing stochastic noise. A difference-of-differences statistic captures disease-specific shifts, with Fisher’s method aggregating significance and enrichment tests identifying cell types overrepresented in patients.

**Results:**

In simulations and real-world datasets, the framework showed strong performance. In large peripheral blood cohorts, it consistently identified trait-associated populations, with monocytes as a benchmark. Analyses of disease datasets revealed both established and novel pathogenic populations, including age-associated B cells and cytotoxic CD8^+^ T cells in systemic lupus erythematosus, antigen-presenting acinar cells in Sjögren’s syndrome, and dysregulated proximal tubules in chronic kidney disease.

**Conclusions:**

These findings demonstrate the utility of integrating GWAS with single-cell transcriptomics to pinpoint disease-relevant cells in complex traits.

